# GLP-1 Receptor Activation Inhibits VLDL Production and Reverses Hepatic Steatosis by Decreasing Hepatic Lipogenesis in High-Fat-Fed APOE*3-Leiden Mice

**DOI:** 10.1371/journal.pone.0049152

**Published:** 2012-11-02

**Authors:** Edwin T. Parlevliet, Yanan Wang, Janine J. Geerling, Janny P. Schröder-Van der Elst, Kristen Picha, Karyn O'Neil, Vedrana Stojanovic-Susulic, Tatiana Ort, Louis M. Havekes, Johannes A. Romijn, Hanno Pijl, Patrick C. N. Rensen

**Affiliations:** 1 Department of General Internal Medicine, Endocrinology and Metabolic Diseases, Leiden University Medical Center, Leiden, The Netherlands; 2 Department of Cardiology, Leiden University Medical Center, Leiden, The Netherlands; 3 Janssen Research & Development, LLC, Pennsylvania, United States of America; 4 Gaubius Laboratory, Netherlands Organization for Applied Scientific Research-Metabolic Health Research, Leiden, The Netherlands; University of Amsterdam Academic Medical Center, The Netherlands

## Abstract

**Objective:**

In addition to improve glucose intolerance, recent studies suggest that glucagon-like peptide-1 (GLP-1) receptor agonism also decreases triglyceride (TG) levels. The aim of this study was to evaluate the effect of GLP-1 receptor agonism on very-low-density lipoprotein (VLDL)-TG production and liver TG metabolism.

**Experimental Approach:**

The GLP-1 peptide analogues CNTO3649 and exendin-4 were continuously administered subcutaneously to high fat diet-fed APOE*3-Leiden transgenic mice. After 4 weeks, hepatic VLDL production, lipid content, and expression profiles of selected genes involved in lipid metabolism were determined.

**Results:**

CNTO3649 and exendin-4 reduced fasting plasma glucose (up to −30% and −28% respectively) and insulin (−43% and −65% respectively). In addition, these agents reduced VLDL-TG production (−36% and −54% respectively) and VLDL-apoB production (−36% and −43% respectively), indicating reduced production of VLDL particles rather than reduced lipidation of apoB. Moreover, they markedly decreased hepatic content of TG (−39% and −55% respectively), cholesterol (−30% and −55% respectively), and phospholipids (−23% and −36% respectively), accompanied by down-regulation of expression of genes involved in hepatic lipogenesis (*Srebp-1c*, *Fasn*, *Dgat1*) and apoB synthesis (*Apob*).

**Conclusion:**

GLP-1 receptor agonism reduces VLDL production and hepatic steatosis in addition to an improvement of glycemic control. These data suggest that GLP-receptor agonists could reduce hepatic steatosis and ameliorate dyslipidemia in patients with type 2 diabetes mellitus.

## Introduction

Type 2 diabetes mellitus (T2DM) has become a major metabolic disorder in both developed and developing countries, with impaired glucose tolerance and insulin resistance as hallmarks [1], [2]. In addition to glucose metabolism, lipid metabolism is disturbed in T2DM patients, reflected by increased plasma levels of low-density lipoprotein, VLDL-TG, and decreased levels of high-density lipoprotein. Moreover, T2DM is strongly associated with fatty liver disease (i.e. hepatic steatosis) [3], for which no effective pharmacotherapeutic options are yet available.

GLP-1 is an incretin hormone produced by intestinal L cells and the brain [4], [5]. GLP-1 is released in response to food intake to stimulate glucose-dependent insulin secretion by the pancreas [4], [6]. Additionally, GLP-1 exerts multiple other effects, including inhibition of food intake [7], slowing gastric emptying [8], and inhibition of glucagon secretion [9]. Thus, GLP-1 was considered as a good target for the treatment of T2DM. However, therapeutic application of GLP-1 is hampered due to its short circulating half-life (<2 minutes), because it is rapidly degraded by dipeptidyl peptidase 4 (DPP-4) that is widely expressed in endothelium and intestinal mucosa [10]. Therefore, pharmaceutical GLP-1 analogues that are resistant to inactivation by DPP-4 have been developed with an improved pharmacokinetic profile related to a longer half-life, of which exenatide (a synthetic version of exendin-4) was approved in 2005 for the treatment of T2DM [11]. We have previously described that CNTO736, a GLP-1 Mimetibody^TM^ receptor agonist that incorporates a GLP-1 peptide analogue genetically fused by a unique linker to a domain that includes the Fc portion of an antibody, has an even longer circulating half-life than exendin-4 and retains the beneficial effects of GLP-1 on glucose metabolism [12]. The long-acting GLP-1 analogue CNTO3649, a more recent version of CNTO736 with two point mutations introduced to improve protein solubility, retains this advantageous pharmacokinetic profile.

In addition to improving glucose metabolism, preliminary studies suggested that GLP-1 receptor agonism decreases plasma TG levels in patients with T2DM [13], [14]. However, the mechanism underlying these beneficial effects on TG metabolism remains unclear. Therefore, the objective of the present study was to evaluate the effects of GLP-1 receptor agonism via CNTO3649 and exendin-4 on VLDL-TG production and liver TG metabolism, and further to explore the underlying mechanisms, in APOE*3-Leiden (*E3L*) transgenic mice fed a high fat diet (HFD) [15].

## Materials and Methods

### Animals

For all experiments, 8–10 weeks old male *E3L* mice [16] were used, housed in a temperature and humidity-controlled environment with free access to food and water. Experiments were performed after 7 h of fasting at 14 ∶00 pm with food withdrawn at 7∶00 am. Body weight was measured weekly during experiments. All animal experiments were performed in accordance with the regulations of Dutch law on animal welfare, and the Institutional Ethics Committee for Animal Procedures from the Leiden University Medical Center, Leiden, The Netherlands, approved the protocol. All surgery was performed under isoflurane anesthesia

### Experiments

Two experiments were conducted, each of which was designed to investigate a specific aspect of the overall hypothesis.

In the first experiment, mice were fed a HFD (44 energy% fat, derived from bovine fat; Hope Farms, Woerden, The Netherlands) for 22 weeks. After 18 weeks of HFD feeding, mice were divided into 5 groups, matched for fasting body weight and plasma glucose levels. An osmotic minipump (model 1004, Alzet DURECT Corp., Cupertino, CA) was implanted subcutaneously in the left back region under light isoflurane anesthesia for the continuous delivery of CNTO3649 (1.0 or 3.0 mg/kg/day, dissolved in PBS), exendin-4 (15 or 50 µg/kg/day, dissolved in PBS) or PBS as a control for up to 4 weeks, while continuously feeding mice the HFD. Additionally, one group of mice received PBS while being fed a chow diet throughout the whole experiment as a control for HFD feeding. After 4 weeks of drug treatment, hepatic VLDL-TG and VLDL-apoB production were determined.

In the second experiment, mice were fed the HFD for 13 weeks. After 9 weeks of HFD feeding, mice were divided into 5 groups, matched for fasting body weight and plasma glucose levels. Osmotic minipumps were implanted subcutaneously for the continuous delivery of CNTO3649 (0.3 or 1.0 mg/kg/day, dissolved in PBS), exendin-4 (15 or 50 µg/kg/day, dissolved in PBS) or PBS as a control for up to 4 weeks, while continuously feeding the mice the HFD. Additionally, one group of mice received PBS while being fed a chow diet as a control for HFD feeding. After 4 weeks of drug treatment, mice were perfused with ice-cold PBS via the heart, and livers were isolated to investigate hepatic lipid content and determine expression of selected genes involved in lipid metabolism. In addition, skeletal muscles from the hind leg were isolated to determine expression of selected genes involved in thermogenesis and fatty acid oxidation.

### Compounds

CNTO3649 (molecular weight  = 68,000 g/mol) was constructed by fusing a GLP-1 peptide analogue to a flexible Gly/Ser linker and a fragment of a V region heavy chain (VH) domain linked directly to the CH_2_ and CH_3_ domains of an Fc as described previously for CNTO736 [17]. Exendin-4 (molecular weight  = 4186.6 g/mol) was purchased from Sigma (St. Louis, MO).

### Plasma glucose and insulin analysis

Blood was collected by tail bleeding into chilled capillary tubes. The tubes were placed on ice and centrifuged, and the obtained plasma was snap-frozen in liquid nitrogen and stored at −20°C for further measurements. Plasma was assayed for glucose using a commercially available enzymatic kit according to the manufacturer's protocol (Instruchemie, Delfzijl, The Netherlands), and insulin was measured by ELISA (Mercodia AB, Uppsala, Sweden).

### Hepatic VLDL-TG and VLDL-apoB production

Mice were fasted for 7 hours, with food withdrawn at 7.00 am and anesthetized by intraperitoneal injection of 6.25 mg/kg acepromazine (Alfasan, Woerden, The Netherlands), 6.25 mg/kg midazolam (Roche, Mijdrecht, The Netherlands), and 0.3125 mg/kg fentanyl (Janssen-Cilag, Tilburg, The Netherlands). Mice received an intravenous (iv) injection of 100 µl PBS containing 100 µCi Tran^35^S label (MP Biomedicals, Eindhoven, the Netherlands) resulting in incorporation of ^35^S into newly produced apoB required for hepatic VLDL production. After 30 min, the animals received an iv injection of tyloxapol (500 mg/kg body weight; Triton WR-1339, Sigma), as a 10% (w/w) solution in sterile saline, to prevent systemic lipolysis of newly secreted hepatic VLDL-TG [18]. Blood samples were drawn before (t = 0) and at 15, 30, 60, and 90 min after tyloxapol injection. Plasma was assayed for TG concentration using the commercially available enzymatic kit 11488872 (Roche Molecular Biochemicals, Indianapolis, IN). At 120 min, mice were euthanized, and blood was collected by orbital puncture for isolation of VLDL by density gradient ultracentrifugation [19]. ^35^S-apoB was measured in the VLDL fraction and VLDL-apoB production rate was calculated as dpm.h^−1^ [20]. TG and total cholesterol (TC) concentrations in the VLDL fractions were determined using the commercially available enzymatic kits 11488872 and 236691 (Roche) respectively, and phospholipid (PL) concentration was measured using a commercial kit (phospholipids B, Wako Chemicals, Neuss, Germany).

### Hepatic lipid content

Liver lipids were extracted according to a modified protocol from Bligh and Dyer [21]. Briefly, small liver pieces were homogenized in ice-cold methanol. After centrifugation, lipids were extracted by addition of 1800 µl CH_3_OH: CHCl_3_ (1∶3 v/v) to 45 µl homogenate, followed by vigorous vortexing and phase separation by centrifugation (5 min at 2,000 rpm). The CHCl_3_ phase was dried and dissolved in 2% Triton X-100. TG, TC, and PL concentrations were measured using commercial kits as described above. Liver lipids were expressed as nmol per mg protein, which was determined using the BCA protein assay kit.

### Hepatic gene expression analysis

Total RNA was extracted from liver pieces using the Nucleospin RNA II kit (Macherey-Nagel, Duren, Germany) or from muscle pieces using the RNeasy Fibrous Tissue Mini kit (Qiagen, Valencia, CA, USA) according to manufacturer's instructions. RNA quality of each sample was examined by the lab-on-a-chip method using Experion Std Sens analysis kit (Biorad, Hercules, CA) and RNA concentration of each sample was determined by Nanodrop technology (Thermo Scientific, Wilmington, USA). Then, total RNA was reverse-transcribed with iScript cDNA synthesis kit (1708891, Bio-Rad), subsequently, obtained cDNA was purified with Nucleospin Extract II kit (636973, Macherey-Nagel, Bioké). Real-time PCR was performed on a CFX96 machine (Bio-Rad), the reaction mixture consisting of SYBR-Green Sensimix (QT615, GC Biotech), cDNA, primers (Biolegio, Nijmegen, The Netherlands), and nuclease-free water in a total reaction volume of 10 µl. mRNA values of each gene were normalized to mRNA levels of cyclophilin (*Cyclo*) and hypoxanthine ribosyltransferase (*Hprt*). Primer sequences are listed in Table S1.

### Statistical analysis

Differences between groups were determined with the Kruskal-Wallis non-parametric test for *k* independent samples. When significant differences were found, the Mann-Whitney non-parametric test was used as a post-hoc test to determine differences between two independent groups. A P-value of less than 0.05 was considered statistically significant. Data are presented as means ± SEM.

## Results

### GLP-1 receptor agonism reduces body weight and fasting plasma glucose and insulin levels in high fat diet-fed *E3L* mice

**Figure 1:**
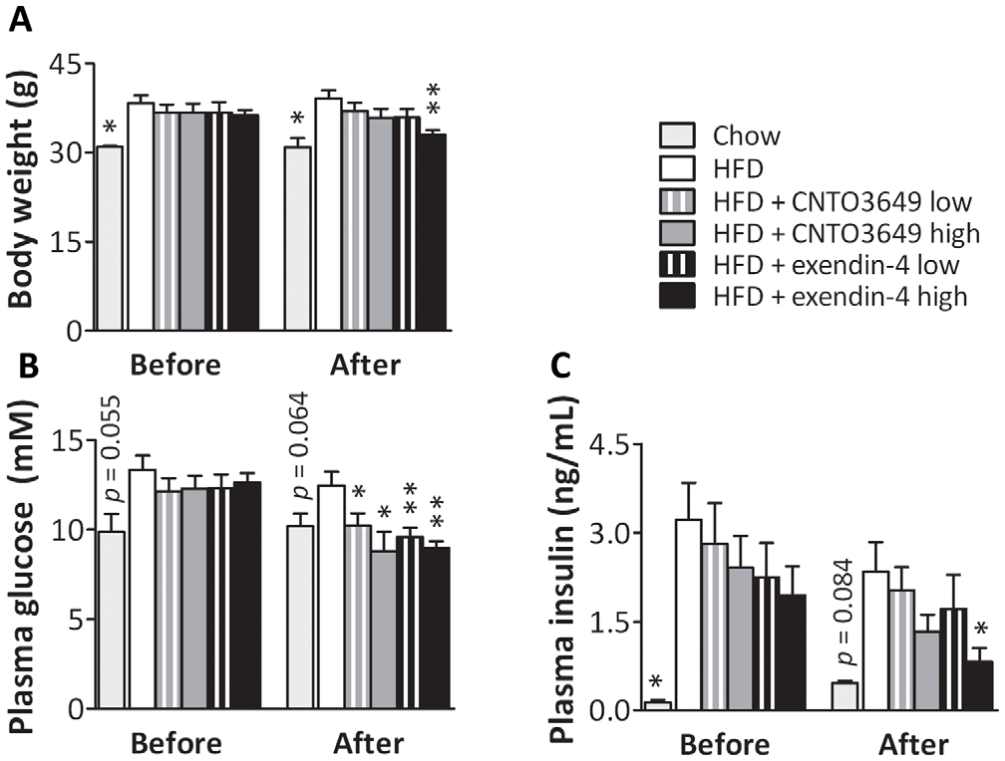
GLP-1 receptor agonism reduces body weight and fasting plasma glucose and insulin levels. APOE*3-Leiden (*E3L*) mice were fed a high fat diet (HFD) for 22 weeks. The last 4 weeks, mice were treated with either vehicle (HFD control), CNTO3649 (1.0 or 3.0 mg/kg/day) or exendin-4 (15 or 50 μg/kg/day). As a control for HFD feeding, an additional group of mice was included fed a chow diet that received vehicle (chow control). Blood was collected by tail bleeding after 7 h of fasting. Just before drug treatment (week 18) and after treatment (week 22), body weight (A), plasma glucose (B) and plasma insulin (C) levels were determined. Values are means ± SEM for at least 6 mice per group. *P<0.05, **P<0.01 compared to HFD controls.

*E3L* mice were fed a HFD for 18 weeks and thereafter were treated with the GLP-1 receptor agonists CNTO3649 or exendin-4 via subcutaneous osmotic minipumps for 4 weeks while continuing the HFD. Body weight and fasting plasma glucose and insulin levels before and after treatment are shown in Figure 1. Eighteen weeks of HFD feeding increased body weight (+24%, P<0.05) (Fig. 1A), tended to increase fasting plasma glucose (+36%, P = 0.055) (Fig. 1B) and increased fasting insulin levels (13-fold, P<0.05) (Fig. 1C) compared to chow diet feeding. CNTO3649 (1.0 and 3.0 mg/kg/day) and the low dose of exendin-4 (15 µg/kg/day) did not affect body weight, whereas the high dose of exendin-4 (50 µg/kg/day) decreased body weight (-16%, P<0.01) (Fig. 1A) as compared to HFD control mice. Both doses of CNTO3649 and exendin-4 decreased fasting plasma glucose levels (up to −30%, P<0.05 and −28%, P<0.01, respectively) compared to HFD control mice (Fig. 1B). Additionally, the high dose of both CNTO3649 and exendin-4 decreased plasma insulin (−43% and −65%, respectively, P<0.05 for exendin-4 only) compared to HFD control mice (Fig. 1C). Collectively, these data confirm that GLP-1 receptor agonism by either CNTO3649 or exendin-4 improved glycemic control in the HFD-fed *E3L* mouse model.

### GLP-1 receptor agonism reduces hepatic secretion of VLDL particles without affecting particle composition

**Figure 2:**
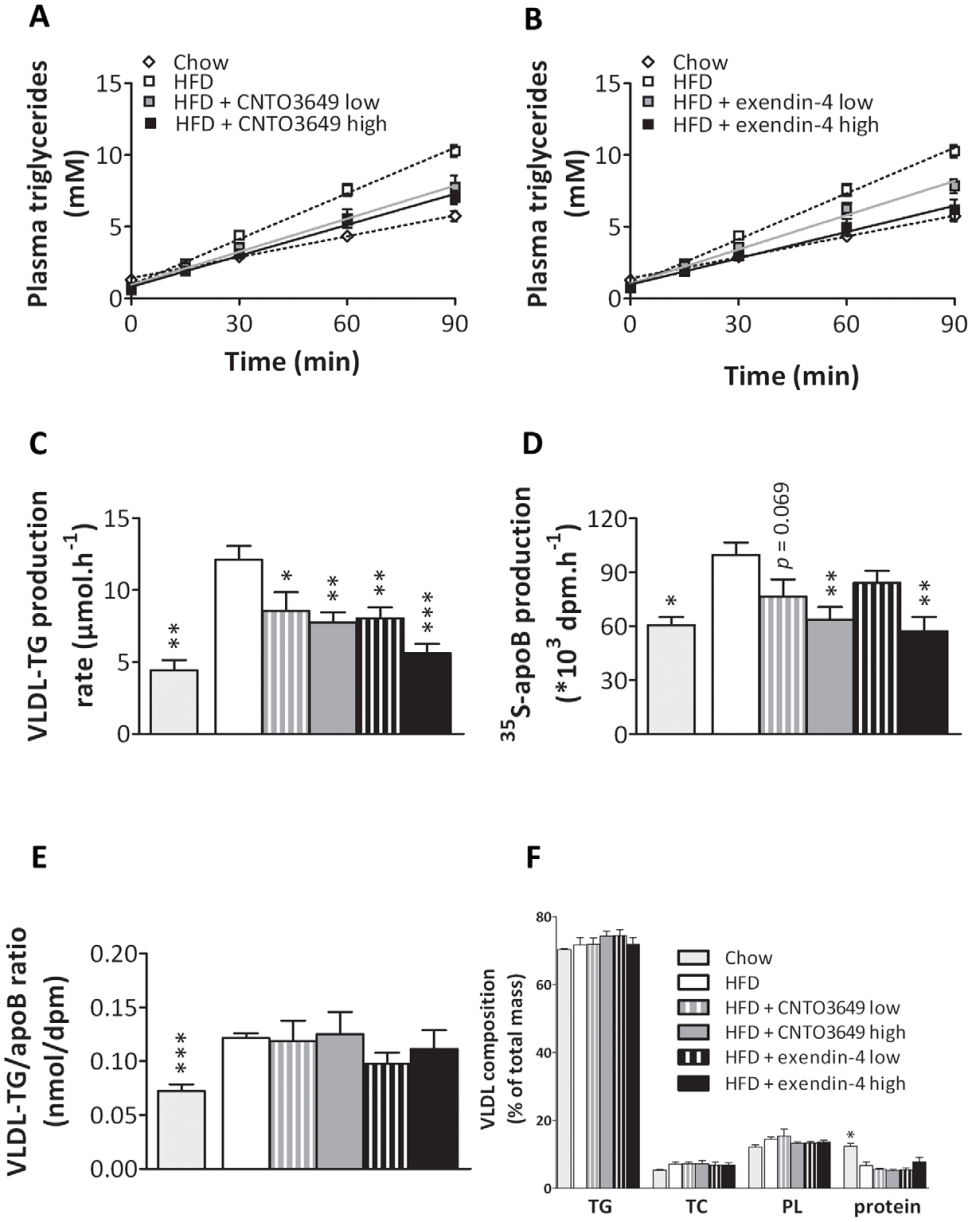
GLP-1 receptor agonism reduces hepatic VLDL-TG and VLDL-apoB production without affecting VLDL particle composition. *E3L* mice were fed a HFD for 22 weeks. The last 4 weeks, mice were treated with either vehicle (HFD control), CNTO3649 (1.0 or 3.0 mg/kg/day) or exendin-4 (15 or 50 μg/kg/day). As a control for HFD feeding, an additional group of mice fed a chow diet was included that received vehicle (chow control). After 7 h fasting, mice were injected with Tran^35^S label (t = −30 min) and Triton WR-1339 (t = 0 min). Blood was drawn at the indicated time points and plasma TG concentrations were determined (A, B). VLDL-TG production rate was calculated as µmol/h from the slopes of the TG-time curves of the individual mice (C). At t = 120 min, mice were exsanguinated, and VLDL was isolated by density gradient ultracentrifugation. ^35^S-activity was determined, and VLDL-apoB production rate was calculated as dpm.h^−1^ (D). The VLDL-TG production rate to VLDL-apoB production rate ratio was calculated as nmol/dpm (E). The content of triglycerides, cholesterol, phospholipids and protein in VLDL was determined and calculated as % of total mass (F). Values are means ± SEM for at least 6 mice per group. *P<0.05, **P<0.01, ***P<0.001 compared to HFD controls. TG: triglycerides; TC: total cholesterol; PL: phospholipids; Pro: protein.

To evaluate the effect of GLP-1 receptor agonism on hepatic VLDL production, mice received an intravenous injection of Tran^35^S to label newly formed apoB, and tyloxapol to block LPL-mediated lipolysis of newly synthesized VLDL. HFD feeding increased the hepatic production rate of both VLDL-TG (Fig. 2A–C) and VLDL-apoB (Fig. 2D) compared to chow diet, which is in line with a previous study [22]. Interestingly, the VLDL-TG production rate induced by HFD was reduced by both doses of CNTO3649 (up to −36%, P<0.01) (Fig. 2A, C) and exendin-4 (up to −54%, P<0.001) (Fig. 2B, C), as determined from the slope of the curve from the individual mice. Likewise, the VLDL-apoB production rate as induced by HFD was decreased by the high dose of both CNTO3649 and exendin-4 (−36% and −43%, P<0.01, respectively) (Fig. 2D). HFD feeding increased the TG/apoB ratio within VLDL as compared to chow feeding by +68% (P<0.001) (Fig. 2E), indicating that HFD induces the formation of larger lipid-enriched VLDL particles. However, both CNTO3649 and exendin-4 did not affect the VLDL-TG/apoB ratio compared with HFD control group. Since each VLDL particle contains a single apoB molecule, GLP-1 receptor agonism apparently decreases the production rate of VLDL particles rather than decreasing the lipidation of VLDL particles. Accordingly, CNTO3649 and exendin-4 did not affect the composition of VLDL with respect to TG, TC, PL, and protein content as compared with HFD control group (Fig. 2F).

### GLP-1 receptor agonism reverses high fat diet-induced hepatic steatosis

**Figure 3:**
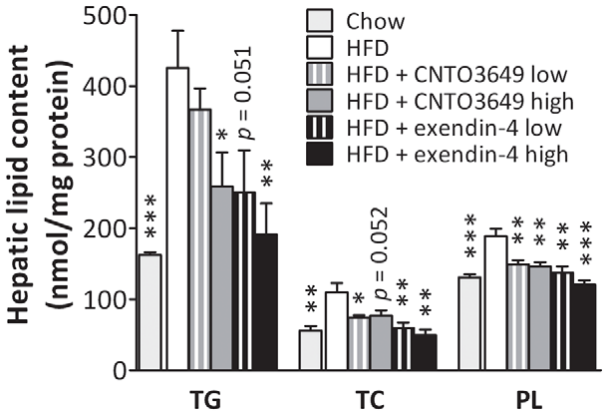
GLP-1 receptor agonism reverses high fat diet-induced hepatic steatosis. *E3L* mice were fed HFD for 13 weeks. The last 4 weeks, mice were treated with either vehicle (HFD control), CNTO3649 (0.3 or 1.0 mg/kg/day) or exendin-4 (15 or 50 μg/kg/day). As a control for HFD feeding, an additional group of mice was included fed a chow diet that received vehicle (chow control). Livers were isolated from 7 h fasted mice, liver pieces were homogenized, and triglycerides, cholesterol and phospholipids were determined as nmol per mg protein. Values are means ± SEM for at least 6 mice per group. *P<0.05, **P<0.01, ***P<0.001 compared to HFD controls.

To obtain insight into the mechanism underlying the reduction in hepatic VLDL production induced by GLP-1 agonism, we next determined the effect of the GLP-1 receptor agonists on hepatic lipid content in a second set of mice. Consistent with the first experiment, CNTO3649 and exendin-4 decreased body weight and fasting plasma glucose and insulin levels compared to the HFD control group (Figure S1) to a similar extent as in the first study. HFD feeding induced a marked increase in hepatic TG, TC, and PL content compared to chow diet (Fig. 3), indicating that HFD leads to hepatic steatosis. The high dose of both CNTO3649 and exendin-4 largely reduced hepatic TG (−39%, P<0.05 and −55%, P<0.01, respectively). Hepatic TC was reduced by both doses of CNTO3649 and exendin-4, (up to −32%, P<0.05 and −55%, P<0.01, respectively). Also, both doses of CNTO3649 and exendin-4 reduced hepatic PL (up to −23%, P<0.01 and −36%, P<0.01, respectively). Importantly, hepatic lipid content observed after treatment with the high dosages of CNTO3649 and exendin-4 group did not differ from that of the chow control group (P>0.05), suggesting that GLP-1 receptor agonism completely reversed HFD-induced hepatic steatosis.

### GLP-1 receptor agonism affects hepatic expression of genes involved in VLDL production, lipogenesis and lipid homeostasis

**Figure 4:**
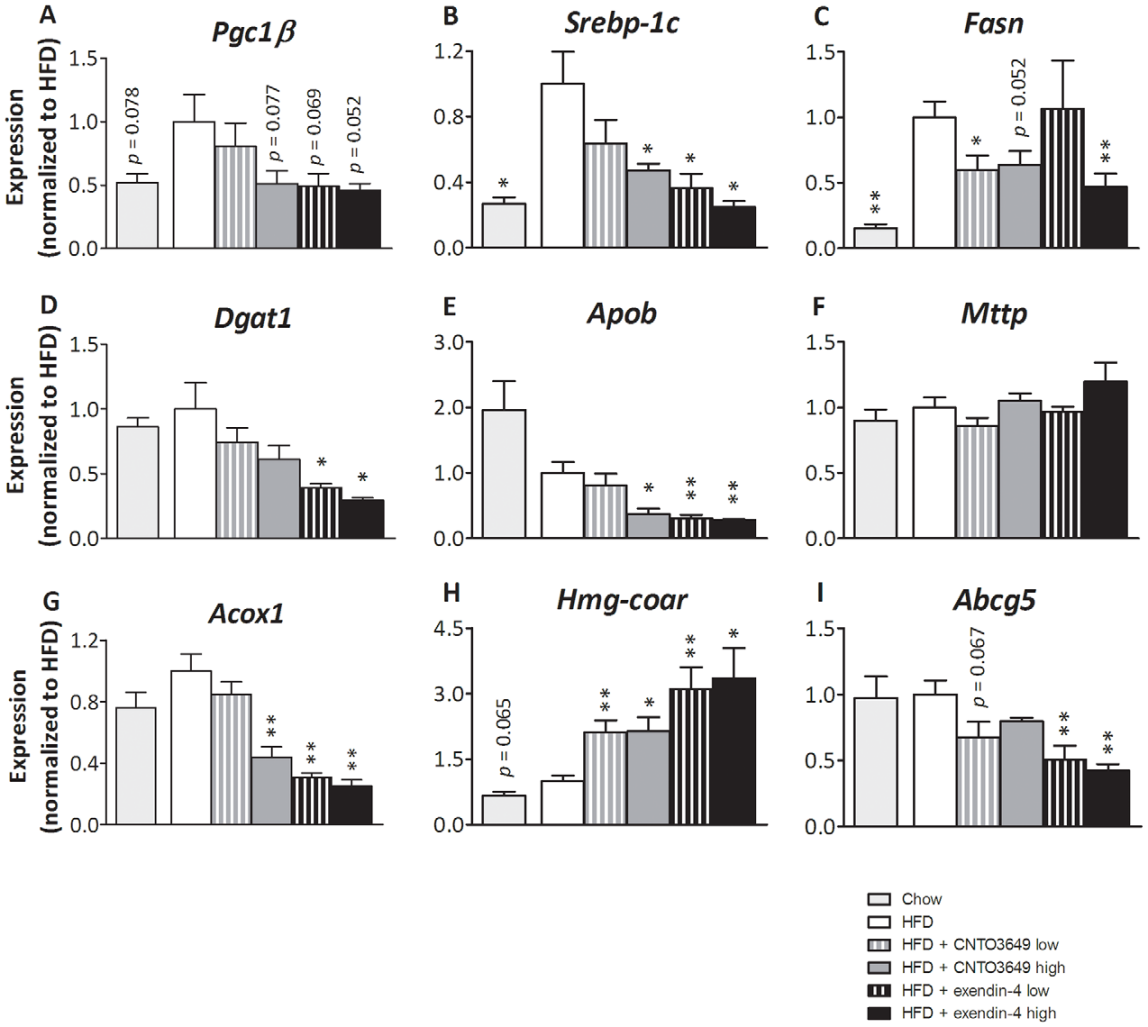
GLP-1 receptor agonism affects hepatic expression of genes involved in VLDL production, lipogenesis, and lipid homeostasis. *E3L* mice were fed HFD for 13 weeks. The last 4 weeks, mice were treated with either vehicle (HFD control), CNTO3649 (0.3 or 1.0 mg/kg/day) or exendin-4 (15 or 50 μg/kg/day). As a control for HFD feeding, an additional group of mice was included fed a chow diet that received vehicle (chow control). Livers were isolated from 7 h fasted mice, and mRNA was extracted from liver pieces. mRNA values of indicated genes were normalized to *Cyclo* and *Hprt* mRNA levels. Data were calculated as fold difference as compared with the HFD control group. Values are means ± SEM for at least 6 mice per group. *P<0.05, **P<0.01, ***P<0.001 compared to HFD controls.

To elucidate the mechanism how GLP-1 receptor agonism reduces liver lipids and VLDL secretion, we investigated the hepatic expression of genes involved in lipid metabolism (i.e. lipogenesis, VLDL secretion, cholesterol metabolism, and FA oxidation) (Fig. 4). HFD feeding strongly tended to increase the expression of peroxisome proliferator-activated receptor gamma coactivator 1-beta (*Pgc1β)*, whereas the high dose of CNTO3649 and both doses of exendin-4 tended to reduce this HFD-induced increase in *Pgc1β*. Next, HFD feeding increased the expression of the lipogenic transcription factor sterol regulatory element binding protein 1c (*Srebp-1c*) (3.7-fold, P<0.05) (Fig. 4A) and its target gene FA synthase *(Fasn*) (6.7-fold, P<0.01) (Fig. 4B), which plays a role in *de novo* lipogenesis, contributing to HFD-induced hepatic steatosis. The high dose of CNTO3649 and both doses of exendin-4 decreased the expression of *Srebp-1c* (−53%, P<0.05, and up to −75%, P<0.05, respectively) (Fig. 4A) and the low dose of CNTO3649 and the high dose of exendin-4 decreased *Fasn* (−40%, P<0.05 and −53%, P<0.01, respectively) (Fig. 4B) compared with the HFD group. Acyl:diacylglycerol transferase 1 *(Dgat1*), which catalyzes the final step in hepatic TG synthesis, was significantly decreased by exendin-4 only (up to −71%, P<0.05) (Fig. 4C). In addition, the high dose of CNTO3649 and both doses of exendin-4 suppressed expression of apoB (*Apob*) (−62%, P<0.05 and up to −72%, P<0.01, respectively) (Fig. 4D), without affecting expression of microsomal TG transfer protein (*Mttp*) (Fig. 4E) that mediates apoB lipidation. In addition, the high dose of CNTO3649 and both doses of exendin-4 suppressed the expression of the FA oxidation gene acyl-CoA oxidase 1 (*Acox1*) (−56% and up to −75%, P<0.01, respectively) (Fig. 4F). Moreover, both doses of CNTO3649 and exendin-4 increased the expression of HMG-CoA reductase (*Hmgcoar*) (up to 2.1-fold and 3.4-fold, P<0.05, respectively) involved in *de novo* cholesterol synthesis (Fig. 4G). Finally, the expression of ATP-binding cassette sub-family G member 5 (*Abcg5*), involved in bile acid secretion, was significantly decreased for both doses of exendin-4 only (up −58%, P<0.01) (Fig. 4H).

Collectively, these data indicate that GLP-1 receptor agonism decreases lipogenesis and apoB synthesis, consequently resulting in suppression of VLDL particle production and a compensatory decrease in hepatic FA oxidation. In addition, the reduction in hepatic cholesterol content results in compensatory mechanisms to increase hepatic cholesterol synthesis and decrease secretion of hepatic cholesterol as bile acids.

### GLP-1 receptor agonism affects muscle expression of genes involved in fatty acid oxidation

To gain insight into the fate of the FA from the diet we also measured muscle expression of genes involved in thermogenesis and FA oxidation (Figure S2). No differences between groups were found for the thermogenic markers uncoupling protein 1 (*Ucp1*) and peroxisome proliferator-activated receptor gamma coactivator 1-alpha (*Pgc1α*), suggesting that both compounds did not affect energy expenditure by the muscle. However, the expression of *Acox1* was significantly increased for the high dose of CNTO3649 and exendin-4 (+33% and +56%, P<0.05, respectively) as compared to HFD controls. Also, the high dose of exendin-4 increased the expression of carnitine palmitoyltransferase 1 (*Cpt1*) (+73%, P<0.05). These data indicate an increased FA oxidation in muscle.

## Discussion

In the present study, we show that GLP-1 receptor agonism both by CNTO3649 and exendin-4 decreases fasting plasma glucose and insulin levels in HFD-fed *E3L* mice. This is in line with earlier reports that GLP-1 and its analogs ameliorate whole-body glucose intolerance in obese animal models [23], [24] and in T2DM patients [25]. More importantly, to our knowledge, this study is the first to demonstrate that CNTO3649 and exendin-4 reduce hepatic VLDL particle production, evidenced by similarly reduced VLDL-TG and VLDL-apoB production rates. In addition, both GLP-1 receptor agonists largely decrease the hepatic lipid content thereby reversing HFD-induced hepatic steatosis.

Increased plasma VLDL-TG levels are a central feature of T2DM, and are mainly caused by increased hepatic VLDL-TG and VLDL-apoB production [26]. We observed that HFD feeding increased VLDL-TG and VLDL-apoB production, and in addition increased the VLDL-TG/apoB ratio. As each VLDL particle contains a single molecule of apoB, VLDL-apoB reflects particle number, whereas VLDL-TG reflects the major lipid constituent of the particle. An increased VLDL-apoB production rate with a concomitantly increased VLDL-TG/apoB ratio thus indicates that HFD feeding not only results in overproduction of VLDL particles but also in the formation of larger lipid-enriched VLDL particles. Both CNTO3649 and exendin-4 reduced the VLDL-TG and VLDL-apoB production rates without affecting the VLDL-TG/apoB ratio, suggesting that GLP-1 agonism reduces the VLDL particle production without affecting the lipidation of VLDL-apoB. This was indeed confirmed by VLDL composition analysis. Notably, we have observed the same effects in WT mice treated with exendin-4 (data not shown), ruling out a possible impact of the genetic background of the *E3L* mice on the treatment outcome.

We also observed that both CNTO3649 and exendin-4 completely reversed HFD-induced hepatic steatosis reflected by largely decreased hepatic TG and TC contents to the low levels observed in chow-fed control mice. This corroborates recent studies showing that prolonged infusion of exendin-4 in *ob/ob* mice reduced hepatic TG accumulation [27]. Hepatic gene expression analysis revealed that the GLP-1 receptor agonists decreased the expression of the nuclear transcription factor *Srebp-1c* and its targets *Fasn* and *Dgat1*, which are involved in *de novo* FA and TG synthesis, respectively. At the same time, the GLP-1 receptor agonists decreased *ApoB* expression without affecting the expression of *Mttp*, of which the gene product MTP is involved in the transfer of TG onto apoB. On the other hand, they decreased the expression of *Acox1* and *Cpt1a* (not shown), both of which are involved in FA oxidation.

Collectively, these data strongly suggest that GLP-1 receptor agonism primarily reduces hepatic lipogenesis, thereby causing a reduction in hepatic TG content, with a compensatory reduction in FA oxidation. Taken together with the concomitantly reduced apoB production, lower hepatic availability of TG results in a reduced production of VLDL particles. It is known that the contribution of *de novo* lipogenesis to total hepatic VLDL secretion strongly increases from 2–5% under healthy conditions up to 25–30% in T2DM patients with hepatic steatosis [28], [29]. Since HFD feeding of *E3L* mice similarly induced hepatic steatosis, the contribution of *de novo* lipogenesis to the increased VLDL production was likely also augmented by HFD feeding, and reversed by GLP-1 receptor agonism concomitant with attenuating hepatic steatosis. Interestingly, the increased expression of *Acox* and *Cpt1* in the muscle suggests an increase in FA oxidation. In addition, by indirect calorimetry (Figure S3) we observed that exendin-4 treatment reduces the respiratory exchange rate, indicating an increased oxidation of fat. Although this effect was transient, it might have contributed to differences in the observed phenotypes after prolonged treatment. Collectively, these data suggest that GLP-1 receptor agonism not only decreases the production of FA, but also upregulates an oxidative pathway in the muscle to deal with the elevated uptake of FA present in the diet.

The mechanism underlying the reduced hepatic cholesterol content is less clear, although it may be expected from the reduced TG content given the tight relationship between hepatic TG and cholesterol levels. The reduction in hepatic cholesterol content evidently results in a compensatory induction of *Hmgcoar*, involved in *de novo* cholesterol synthesis, and downregulation of *Abcg5*, involved in the elimination of hepatic cholesterol as bile acids into the bile.

It is interesting to speculate on the molecular mechanisms that underlie the reduction in hepatic lipogenesis as induced by GLP-1 receptor agonism. Since *Srebp-1c* plays a crucial role in insulin-mediated *de novo* lipogenesis in the liver [30], it is well possible that the improved HFD-induced glucose intolerance accompanied by reduced insulin levels resulted in downregulation of hepatic *Srebp-1c* expression, thereby attenuating the HFD-induced increase in *de novo* lipogenesis. Beside insulin levels, *Pgc1β* could also be involved as it impacts on *Srebp-1c* expression [31]. Therefore, the observed trend towards a reduction in *Pgc1β* expression might also have contributed to a decreased *Srebp-1c* and consequently a decrease in *de novo* lipogenesis. Interestingly, it has recently been established that human hepatocytes [32], [33] as well as rodent hepatocytes [34] express GLP-1 receptors. In fact, incubation of hepatocytes with GLP-1 and exendin-4 in the absence of insulin directly reduces *Srebp-1c* [34], and reduces hepatocyte steatosis [32]. This indicates that GLP-1 receptor agonism may directly downregulate *Srebp-1c* and lipogenesis through binding of hepatocytic receptors. Third, GLP-1 receptor agonism by exendin-4 or the DPP-4 inhibitor sitagliptin reduces the intestinal production of chylomicron-TG and apoB in hamsters [35], and the DPP-4 inhibitor vildagliptin reduces postprandial chylomicron-TG and apoB in T2DM patients [36]. Indeed, pilot data from our lab confirmed that exendin-4 reduces postprandial TG excursion in mice (unpublished). Since uptake of TG from the diet eventually contributes to VLDL-TG production [37], [38], reduced chylomicron production may contribute to the observed effect of GLP-1 receptor agonism on hepatic VLDL production. Finally, circulating GLP-1 can cross the blood-brain barrier [39] and GLP-1 receptors are abundantly expressed in many brain areas [40]. Several studies have shown that central GLP-1 receptor signaling mediates the effect of GLP-1 on hepatic glucose output [41] and lipid deposition in white adipose tissue [42]. It is therefore reasonable to postulate that the brain-nerve-liver axis might contribute to the beneficial effects of GLP-1 agonism on hepatic lipid metabolism and VLDL secretion.

In our previous study, in which we administered exendin-4 by daily intraperitoneal injections, we were unable to detect any effect of exendin-4 on VLDL production, albeit that exendin-4 did improve glucose tolerance [12]. In that study, daily injections of CNTO736, a previous version of CNTO3649, with a considerably longer half-life than exendin-4, did reduce VLDL production. Since we now demonstrate that continuous delivery of exendin-4 inhibits VLDL production, it is likely that the ability of GLP-1 agonists to reduce hepatic steatosis and VLDL production is mainly determined by their pharmacokinetic profile. Whereas pulsated exposure of GLP-1 (analogues) is sufficient to improve glucose intolerance, more chronic exposure is required for additional beneficial effects on TG metabolism.

How do the present data obtained in *E3L* mice translate to clinical practice? Recently, it has been reported that treatment of T2DM patients with exenatide on top of pioglitazone resulted in a greater decrease in both hepatic TG and plasma TG levels compared to treatment with pioglitazone only [43]. Based on our present data, it is likely that these observations can be explained by a reduction in *Srebp-1c*- induced lipogenesis, resulting in attenuation of the hepatic TG content, reduction of VLDL-TG production and thus plasma VLDL-TG levels. Therefore, it would be interesting to determine the effect of GLP-1 analogues and DPP-4 inhibitors on VLDL production in future human intervention studies. In addition, we anticipate that the effects of long-circulating GLP-1 receptor agonists such as liraglutide (duration of action ≥24 hours) on lipid metabolism will prove to be superior to those of exenatide (duration of action <24 hours) and in addition will lead to better tolerability due to the necessity of injecting once daily only as compared to twice daily for exenatide [44]. In addition to the beneficial effects on glucose metabolism, VLDL secretion and hepatic lipid content, GLP-1 receptor agonism also reduces blood pressure and the severity of myocardial infarction, while it concomitantly improves left ventricular ejection fraction after infarction [45], enforcing GLP-1 receptor agonism as a valuable therapy to combat T2DM and associated cardiovascular diseases.

In conclusion, our results show that GLP-1 agonism not only decreases bodyweight and improves glycemic control, but also reduces HFD-induced hepatic steatosis, thereby reducing hepatic VLDL biosynthesis and secretion. Therefore, we anticipate that GLP-1 receptor agonism is a valuable strategy to treat T2DM patients, especially those with disturbed lipid metabolism related to hepatic steatosis.

## Supporting Information

Figure S1 **GLP-1 receptor agonism reduces fasting glucose and insulin levels.** *E3L* mice were fed a high fat diet (HFD) for 13 weeks. The last 4 weeks, mice were treated with either vehicle (HFD control), CNTO3649 (0.3 or 1.0 mg/kg/day) or exendin-4 (15 or 50 μg/kg/day). As a control for HFD feeding, an additional group of mice fed a chow diet was included that received vehicle (chow control). Just before drug treatment (week 13) and after treatment (week 17), body weight (A), plasma glucose (B) and plasma insulin (C) levels were determined. Values are means ± SEM for at least 6 mice. *P<0.05, **P<0.01, ***P<0.001 compared to HFD controls.
(TIF)

Figure S2 **GLP-1 receptor agonism affects muscle expression of genes involved in fatty acid oxidation.** *E3L* mice were fed HFD for 13 weeks. The last 4 weeks, mice were treated with either vehicle (HFD control), CNTO3649 (0.3 or 1.0 mg/kg/day) or exendin-4 (15 or 50 μg/kg/day). As a control for HFD feeding, an additional group of mice was included fed a chow diet that received vehicle (chow control). Skeletal muscles were isolated from 7 h fasted mice, and mRNA was extracted from muscle pieces. mRNA values of indicated genes were normalized to *Cyclo* and *Hprt* mRNA levels. Data were calculated as fold difference as compared with the HFD control group. Values are means ± SEM for at least 6 mice per group. *P<0.05, **P<0.01 compared to HFD controls.
(TIF)

Figure S3 **Exendin-4 treatment reduces respiratory exchange ratio.** C57Bl/6 mice were fed a HFD for 3 weeks before they were treated with either vehicle (control) or exendin-4 (50 μg/kg/day). Directly after the initiation of the treatment, indirect calorimetry measurements were started. Individual energy intake (A), activity (B), O_2_ consumption, and CO_2_ production rates were monitored. Respiratory exchange rate (C) and total energy expenditure (D) were calculated from the O_2_ consumption and CO_2_ production rates. Lines represent the mean values of 8 mice treated with vehicle (solid lines) or exendin-4 (dotted lines). Black areas under the x-axis represent the dark (12 hours) and white areas the light periods (12 hours). *P<0.05 compared to HFD controls.
(TIF)

Table S1 **Primer sequences used for RT-qPCR.** *Abcg5,* ATP-binding cassette sub-family G member 5; *Acox1,* acyl-CoA oxidase 1; *Apob,* apolipoprotein B; *Cpt1,* carnitine palmitoyltransferase 1; *Cyclo,* cyclophilin; *Dgat1,* acyl:diacylglycerol transferase 1; *Fasn,* fatty acid synthase; *Hmgcoar,* HMG-CoA reductase;. *Hprt,* hypoxanthine ribosyltransferase; *Mttp,* microsomal TG transfer protein; *Pgc1α,* peroxisome proliferator-activated receptor gamma coactivator 1-alpha; *Pgc1β,* peroxisome proliferator-activated receptor gamma coactivator 1-beta; *Srebp-1c,* sterol regulatory element binding protein 1c; *Ucp1,* uncoupling protein 1.
(DOC)
