# Supplementary figures and images for: GLP-1 Receptor Activation Inhibits VLDL Production and Reverses Hepatic Steatosis by Decreasing Hepatic Lipogenesis in High-Fat-Fed APOE*3-Leiden Mice
Source: PLoS One. 2012 Nov 2;7(11):e49152. doi: 10.1371/journal.pone.0049152 (PMC3487842; doi:10.1371/journal.pone.0049152)

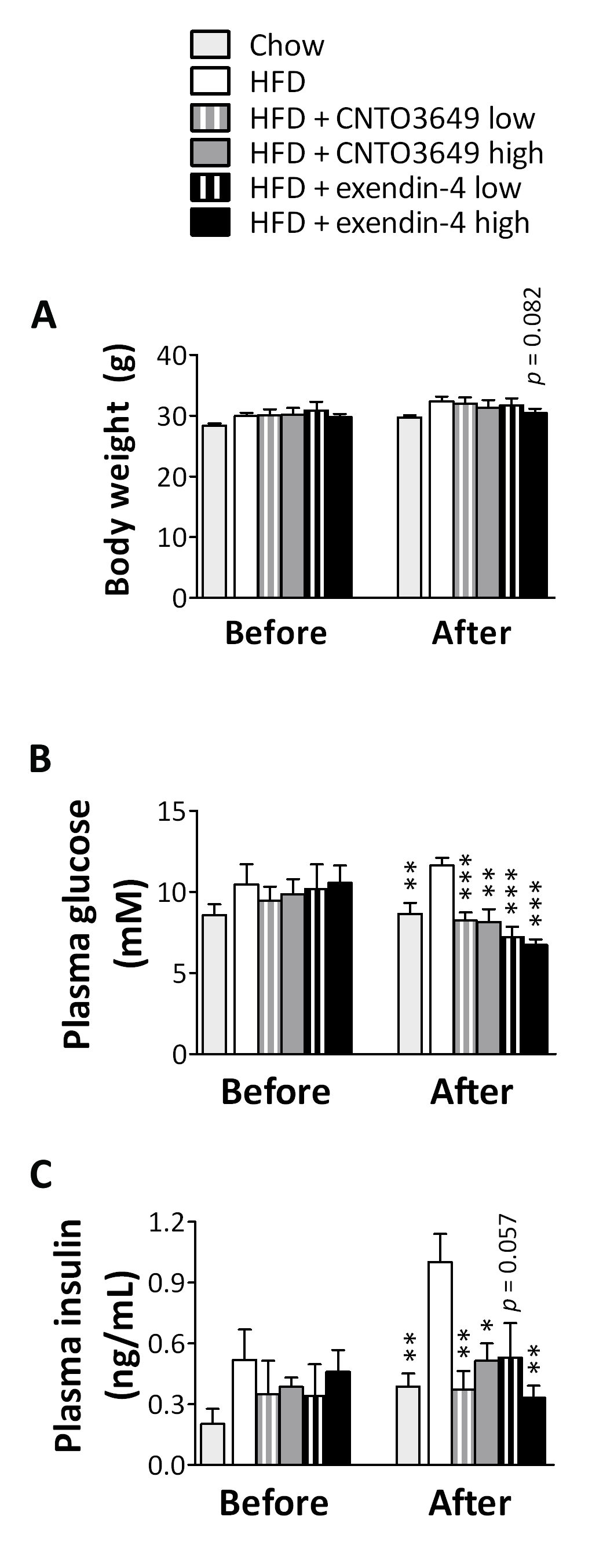

Supplement: Figure S1 — GLP-1 receptor agonism reduces fasting glucose and insulin levels. E3L mice were fed a high fat diet (HFD) for 13 weeks. The last 4 weeks, mice were treated with either vehicle (HFD control), CNTO3649 (0.3 or 1.0 mg/kg/day) or exendin-4 (15 or 50 μg/kg/day). As a control for HFD feeding, an additional group of mice fed a chow diet was included that received vehicle (chow control). Just before drug treatment (week 13) and after treatment (week 17), body weight (A), plasma glucose (B) and plasma insulin (C) levels were determined. Values are means ± SEM for at least 6 mice. *P<0.05, **P<0.01, ***P<0.001 compared to HFD controls. (TIF) [file pone.0049152.s001.tif]

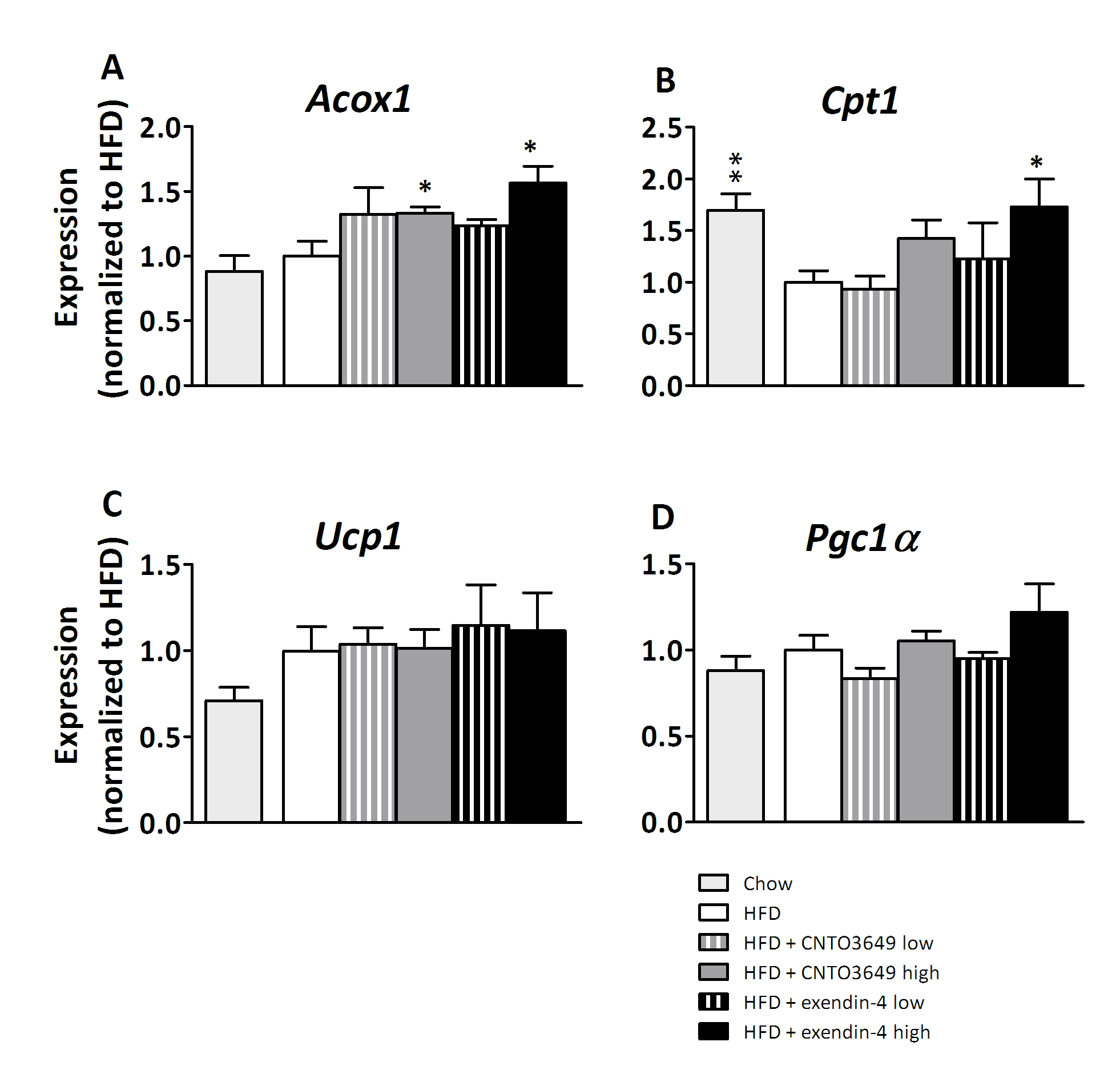

Supplement: Figure S2 — GLP-1 receptor agonism affects muscle expression of genes involved in fatty acid oxidation. E3L mice were fed HFD for 13 weeks. The last 4 weeks, mice were treated with either vehicle (HFD control), CNTO3649 (0.3 or 1.0 mg/kg/day) or exendin-4 (15 or 50 μg/kg/day). As a control for HFD feeding, an additional group of mice was included fed a chow diet that received vehicle (chow control). Skeletal muscles were isolated from 7 h fasted mice, and mRNA was extracted from muscle pieces. mRNA values of indicated genes were normalized to Cyclo and Hprt mRNA levels. Data were calculated as fold difference as compared with the HFD control group. Values are means ± SEM for at least 6 mice per group. *P<0.05, **P<0.01 compared to HFD controls. (TIF) [file pone.0049152.s002.tif]

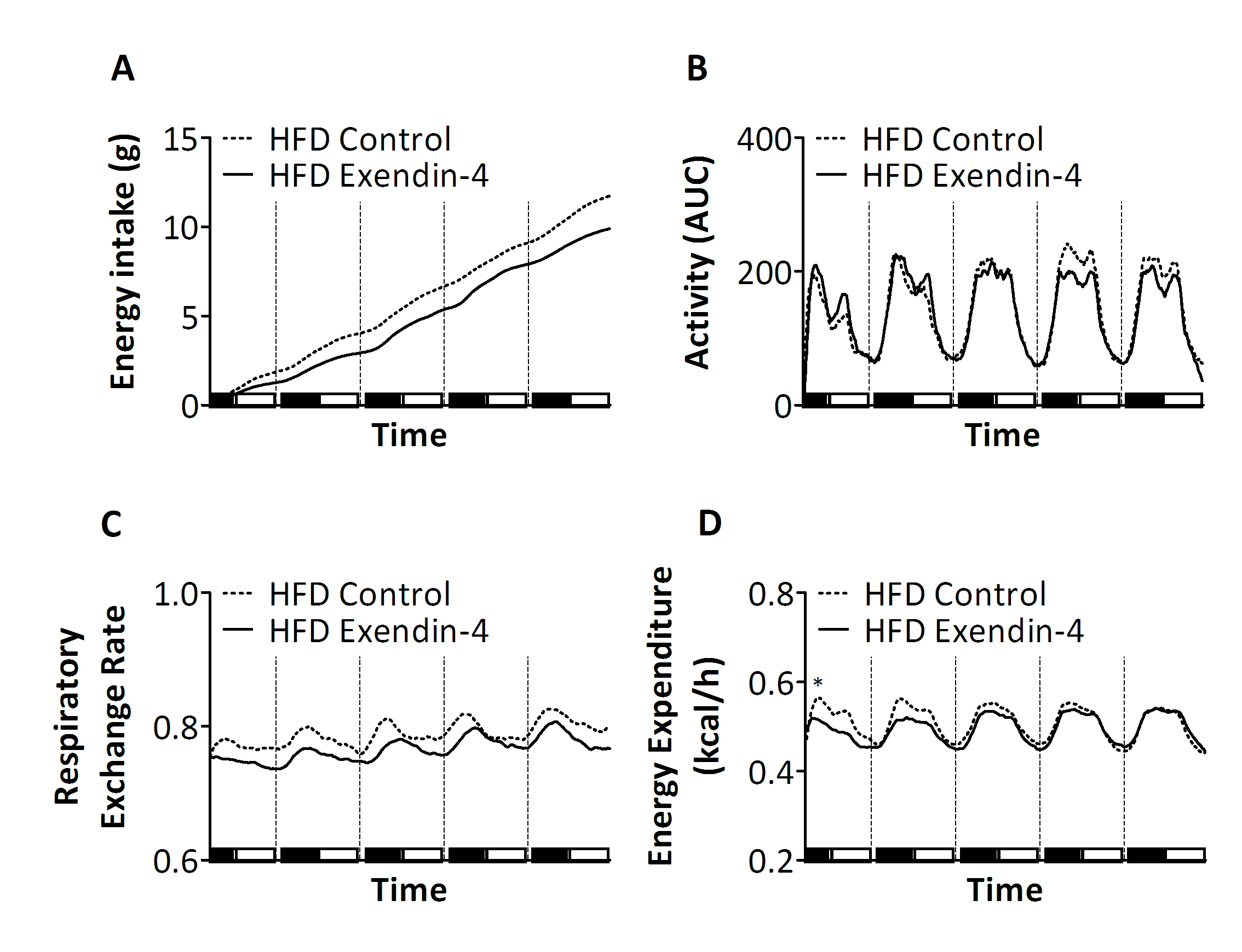

Supplement: Figure S3 — Exendin-4 treatment reduces respiratory exchange ratio. C57Bl/6 mice were fed a HFD for 3 weeks before they were treated with either vehicle (control) or exendin-4 (50 μg/kg/day). Directly after the initiation of the treatment, indirect calorimetry measurements were started. Individual energy intake (A), activity (B), O2 consumption, and CO2 production rates were monitored. Respiratory exchange rate (C) and total energy expenditure (D) were calculated from the O2 consumption and CO2 production rates. Lines represent the mean values of 8 mice treated with vehicle (solid lines) or exendin-4 (dotted lines). Black areas under the x-axis represent the dark (12 hours) and white areas the light periods (12 hours). *P<0.05 compared to HFD controls. (TIF) [file pone.0049152.s003.tif]
