# Supplementary material for: GLP-1 Receptor Activation Inhibits VLDL Production and Reverses Hepatic Steatosis by Decreasing Hepatic Lipogenesis in High-Fat-Fed APOE*3-Leiden Mice
Source: PLoS One. 2012 Nov 2;7(11):e49152. doi: 10.1371/journal.pone.0049152 (PMC3487842; doi:10.1371/journal.pone.0049152)
Supplement: Table S1 — Primer sequences used for RT-qPCR. Abcg5, ATP-binding cassette sub-family G member 5; Acox1, acyl-CoA oxidase 1; Apob, apolipoprotein B; Cpt1, carnitine palmitoyltransferase 1; Cyclo, cyclophilin; Dgat1, acyl:diacylglycerol transferase 1; Fasn, fatty acid synthase; Hmgcoar, HMG-CoA reductase;. Hprt, hypoxanthine ribosyltransferase; Mttp, microsomal TG transfer protein; Pgc1α, peroxisome proliferator-activated receptor gamma coactivator 1-alpha; Pgc1β, peroxisome proliferator-activated receptor gamma coactivator 1-beta; Srebp-1c, sterol regulatory element binding protein 1c; Ucp1, uncoupling protein 1. (DOC) [file pone.0049152.s004.doc]

**Table S1. Primer sequences used for RT-qPCR**

| **Gene** | **Forward primer** | **Reverse Primer** |
| --- | --- | --- |
| *Abcg5* | TGTCCTACAGCGTCAGCAACC | GGCCACTCTCGATGTACAAGG |
| *Acox1* | TATGGGATCAGCCAGAAAGG | ACAGAGCCAAGGGTCACATC |
| *Apob* | GCCCATTGTGGACAAGTTGAT C | CCAGGACTTGGAGGTCTTGGA |
| *Cpt1* | GAGACTTCCAACGCATGACA | ATGGGTTGGGGTGATGTAGA |
| *Cyclo* | CAAATGCTGGACCAAACACAA | GCCATCCAGCCATTCAGTCT |
| *Dgat1* | TCCGTCCAGGGTGGTAGTG | TGAACAAAGAATCTTGCAGACGA |
| *Fasn* | TCCTGGGAGGAATGTAAACAGC | CACAAATTCATTCACTGCAGCC |
| *Hmgcoar* | CCGGCAACAACAAGATCTGTG | ATGTACAGGATGGCGATGCA |
| *Hprt* | TTGCTCGAGATGTCATGAAGGA | AGCAGGTCAGCAAAGAACTTATAG |
| *Mttp* | CTCTTGGCAGTGCTTTTTCTCT | GAGCTTGTATAGCCGCTCATT |
| *Pgc1α* | TGCTAGCGGTTCTCACAGAG | AGTGCTAAGACCGCTGCATT |
| *Pgc1β* | TTGTAGAGTGCCAGGTGCTG | CCTCCATAGCTCAGGTGGAA |
| *Srebp-1c* | GGAGCCATGGATTGCACATT | GGCCCGGGAAGTCACTGT |
| *Ucp1* | TCAGGATTGGCCTCTACGAC | TGCATTCTGACCTTCACGAC |

*Abcg5,* ATP-binding cassette sub-family G member 5; *Acox1,* acyl-CoA oxidase 1; *Apob,* apolipoprotein B; *Cpt1,* carnitine palmitoyltransferase 1; *Cyclo,* cyclophilin; *Dgat1,* acyl:diacylglycerol transferase 1; *Fasn,* fatty acid synthase; *Hmgcoar,* HMG-CoA reductase;. *Hprt,* hypoxanthine ribosyltransferase; *Mttp,* microsomal TG transfer protein; *Pgc1α,* peroxisome proliferator-activated receptor gamma coactivator 1-alpha; *Pgc1β,* peroxisome proliferator-activated receptor gamma coactivator 1-beta; *Srebp-1c,* sterol regulatory element binding protein 1c; *Ucp1,* uncoupling protein 1.
